# Dermatomyositis and antisynthetase syndrome disease activity is associated with the expansion of peripheral GZMB^+^CD4^+^ cytotoxic T cells

**DOI:** 10.3389/fimmu.2026.1863690

**Published:** 2026-07-06

**Authors:** Qin Zhang, Rui Chi, Guoxun Shi, Jiawei Wan, Tianzhi Chang, Yingying Jiang, Wentao Wang, Jing Han, Xiangjie Chen, Fang Gong

**Affiliations:** 1 Department of Laboratory Medicine, Jiangnan University Medical Center (Wuxi No. 2 People’s Hospital), Jiangnan University, Wuxi, Jiangsu, China; 2 Wuxi School of Medicine, Jiangnan University, Wuxi, Jiangsu, China; 3 Department of Rheumatology, Jiangnan University Medical Center (Wuxi No. 2 People’s Hospital), Wuxi, Jiangsu, China

**Keywords:** antisynthetase syndrome, autoimmunity, biomarker, CD4^+^ CTLs, dermatomyositis

## Abstract

**Background:**

Dermatomyositis (DM) and antisynthetase syndrome (ASS) are heterogeneous subsets of idiopathic inflammatory myopathies with persistent unmet needs in disease activity assessment and stratification. Although CD4^+^ T cells are recognized as important contributors to disease pathogenesis, the role of CD4^+^ cytotoxic T lymphocytes (CTLs) remains insufficiently defined.

**Methods:**

Peripheral blood samples were collected from patients with DM (n = 50), patients with ASS (n = 14), and healthy donors (HDs, n = 20). The frequency of CD4^+^ cytotoxic T lymphocytes with expression of cytotoxic protein granzyme B (GZMB^+^CD4^+^ CTLs) was analyzed by flow cytometry to quantify GZMB^+^CD4^+^ CTLs and characterize their phenotype. Disease activity and severity were evaluated using standard clinical indices. Multiplex immunohistochemistry was performed on skin biopsy specimens from patients with DM (n = 4) to assess tissue infiltration and endothelial injury.

**Results:**

The proportion of circulating GZMB^+^CD4^+^ CTLs was significantly increased in both DM and ASS compared with HDs. In patients, this population showed phenotypic features consistent with enhanced cytotoxicity and tissue trafficking, including increased fibroblast growth factor binding protein 2 (FGFBP2) expression and reduced circulating fractions of cells positive for C-X3-C motif chemokine receptor 1 (CX3CR1) and C-X-C motif chemokine receptor 3 (CXCR3). GZMB^+^CD4^+^ CTL frequency was positively associated with global disease activity and cutaneous activity, and decreased after treatment independently of specific drug regimens. Receiver operating characteristic analysis showed that circulating GZMB^+^CD4^+^ CTLs effectively distinguished active from remitting disease in both DM and ASS. In DM skin lesions, GZMB^+^CD4^+^ CTLs were abundant and were accompanied by increased endothelial apoptosis and enhanced HLA-DR expression.

**Conclusions:**

Peripheral GZMB^+^CD4^+^ CTLs are expanded in DM and ASS and are closely associated with disease activity. Their accumulation in lesional tissue and association with endothelial injury support a potential pathogenic role, while their diagnostic performance suggests that they may serve as a biomarker and potential therapeutic target in these diseases.

## Graphical Abstract

**Figure:**
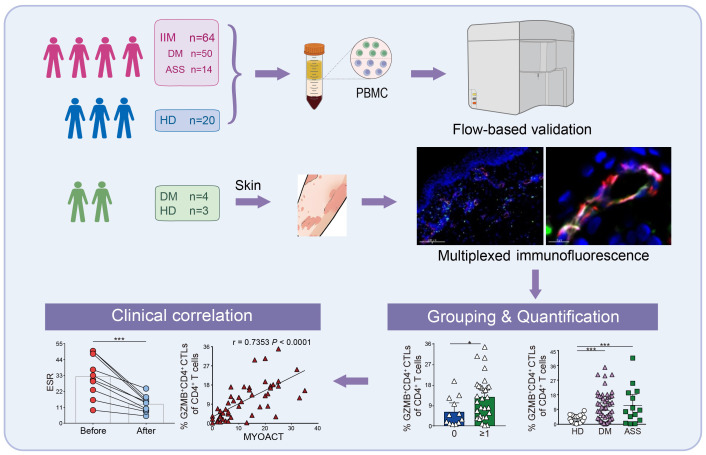

## 1 Introduction

Dermatomyositis (DM) and antisynthetase syndrome (ASS), as subsets of idiopathic inflammatory myopathies (IIM), are chronic, heterogeneous autoimmune diseases characterized by multi-organ involvement, including skin and striated muscles, lungs, joints, gastrointestinal tract and heart (1, 2). Their precise pathogenesis is still incompletely understood, and controversy persists regarding whether ASS is a separate entity or a DM subset (3). Current therapeutic strategies for IIM predominantly involve the use of glucocorticoids and immunosuppressive agents; however, these treatments are frequently constrained by adverse effects and therapeutic resistance (4). Thus, it is essential to gain deeper insights into their pathogenesis in order to develop innovative diagnostic and therapeutic approaches for DM and ASS (1).

Emerging evidence implicates a dysregulation of both innate and adaptive immune responses in the onset and progression of these diseases (5–7). Within affected muscle tissue, CD4^+^ T cells are the predominant infiltrating lymphocyte population and are recognized as key drivers of sustained inflammation in DM (8). An imbalance between Th1 and Th2 cells, with a shift toward a Th2-dominant profile, has been observed during active DM progression (9). Beyond the Th1/Th2 paradigm, patients with ASS exhibit an increased frequency of Th17 cells and a correspondingly elevated Th17/Treg ratio in peripheral blood (10). Conversely, a reduction in Treg frequency has been reported in DM, although this finding was not significant in patients with ASS-associated interstitial lung disease (10, 11).

In recent years, a functionally distinct CD4^+^ T cell subset—CD4^+^ cytotoxic T lymphocytes (CTLs)—has attracted increasing attention. Unlike conventional T helper cells, CD4^+^ CTLs exert antigen-specific cytotoxicity through MHC class II recognition. They subsequently induce target cell lysis via granzyme, perforin, and granulysin-dependent pathways (12). These cells are expanded in the peripheral blood or accumulate in diseased tissues in various autoimmune conditions, including rheumatoid arthritis (13), multiple sclerosis (14, 15), and inflammatory bowel disease (16). Notably, in polymyositis, CD4^+^ CTLs mediate perforin-dependent autologous muscle destruction. These proinflammatory, perforin-expressing cells correlate with disease activity. IFN-γ from CD4^+^ CTLs upregulates muscle HLA class II expression, exacerbating cytotoxic injury. CD4^+^ CTLs are thus key drivers of myositis-related muscle damage (17–19). Nonetheless, the role of CD4^+^ CTLs in the progression of DM and ASS remains largely unexplored.

Here in this study, we observed a marked increase in the proportion of granzyme B-positive CD4^+^ cytotoxic T lymphocytes (GZMB^+^CD4^+^ CTLs) in patients with DM and ASS and found a positive correlation with disease activity. This correlation positions GZMB^+^CD4^+^ CTLs as a potential biomarker for monitoring disease activity. Our findings further reveal that GZMB^+^CD4^+^ CTLs possess the ability to migrate towards and infiltrate lesional tissues, facilitating endothelial cell apoptosis and ultimately contributing to disease progression.

## 2 Materials and methods

### 2.1 Ethical statement

The Ethics Committee of Jiangnan University Medical Center (Wuxi, China) reviewed and approved this project (Approval No. 2025 Ethics Review Y-68). All participants provided written informed consent prior to enrollment. All procedures complied with the Declaration of Helsinki.

### 2.2 Human specimens

Peripheral blood and skin tissue specimens were collected from inpatients at the Department of Rheumatology and Immunology, Jiangnan University Medical Center. Patients were classified as DM or ASS according to clinical diagnosis and predominant clinical phenotype assessed by rheumatologists. DM was classified with reference to the 2017 EULAR/ACR classification criteria for IIMs and their major subgroups (20). Patients were assigned to the DM group when the predominant phenotype was compatible with dermatomyositis, particularly when typical DM-related cutaneous manifestations were present, including Gottron’s sign/papules, heliotrope or periorbital edema, V sign, shawl sign, photosensitive rash, ulcerative or necrotic skin lesions, together with compatible muscle involvement and laboratory findings. Patients were classified as having ASS if at least one anti-aminoacyl-tRNA synthetase (anti-ARS) antibody was detected and at least one ASS-related clinical manifestation was present, including interstitial lung disease, inflammatory myopathy, arthritis, Raynaud’s phenomenon, mechanic’s hands, or fever (21, 22). Individuals with chronic infection, autoimmune disease, or neoplasia were excluded. Demographic data, laboratory results, clinical manifestations, and treatment regimens were collected and recorded (**Supplementary Table 1**).

A certified rheumatologist conducted all clinical assessments. Global disease activity was evaluated using the Myositis Disease Activity Assessment Tool (MYOACT); Global disease damage was assessed using the Myositis Damage Index (MDI); Muscle strength was measured using the Manual Muscle Testing (MMT-8); Skin involvement was evaluated using the Cutaneous Dermatomyositis Disease Area and Severity Index (CDASI). The CDASI score comprises distinct domains for activity and damage. Specifically, skin disease activity was evaluated using the CDASI activity component (CDASI-A). CDASI-A values were classified as low (<19 points), moderate (19–34 points), or high (>34 points) (23–26). Peripheral blood samples were collected during clinically indicated evaluations, including initial presentation, hospitalization, and follow-up. Samples before treatment were defined as those collected before systemic treatment, either at initial presentation or at disease relapse after complete symptom remission. Samples after treatment were defined as those collected after medication when the patient’s symptoms had clinically improved or resolved. Additionally, the type of immunosuppressive therapy administered was documented. Complete clinical remission was defined as the absence of both muscular and extra-muscular disease activity for a minimum duration of six months, either during ongoing immunosuppressive treatment or following the cessation of treatment (27).

### 2.3 Blood sample preparation

Peripheral blood sample was collected into EDTA-2K tubes and processed to obtain peripheral blood mononuclear cell (PBMC) and plasma isolation. Plasma was separated by centrifugation (500 × g for 5 minutes at 4°C), carefully collected, and stored at -80 °C until analysis. Before density gradient separation, the blood was diluted 1:1 with normal saline (0.9% NaCl). The diluted samples were layered onto Ficoll-Hypaque (Cytiva) and centrifuged at 500 × g for 25 minutes at 20°C with the brake turned off. The PBMC layer was collected carefully and washed twice with normal saline. After centrifugation, cells were resuspended in fetal bovine serum supplemented with 10% DMSO (Sigma). Aliquots of cells were quickly transferred to a controlled-rate freezing container at -80°C overnight. Samples were cryopreserved in liquid nitrogen for long-term storage.

### 2.4 Flow cytometry

PBMCs were first stained with Zombie Violet™ viability dye (BioLegend) to discriminate viable cells from nonviable cells. An Fc-receptor blocking step was then applied (1:5 dilution, Miltenyi Biotec) to minimize nonspecific binding. Cells were then stained with chemokine receptor antibodies. After 10 minutes incubation at 37 °C in the dark, cells were washed twice in FACS buffer and then resuspended in 50 μL FACS buffer. For surface staining, cells were stained with mixed fluorochrome-conjugated antibodies for 30 min at 4 °C. For intracellular staining, cells were fixed and permeabilized with a transcription factor Buffer Set (Cat: 00-5523, Thermo Fisher Scientific) and intracellularly stained with appropriates fluorochrome labelled antibodies in the Permeabilization buffer for an hour at room temperature. Cells were kept on ice until acquisition. Detailed antibodies used for flow cytometry are listed in **Supplementary Table 2**. Data acquisition was performed on a Cytek Aurora flow cytometer and results were analyzed using FlowJo version 10.0.0.

The following antibodies were used in the study: surface markers included AF532 anti-human CD3 (Cat: 58-0038-42, Clone: UCHT1, eBioscience), BB515 anti-human CD19 (Cat: 564456, Clone: HIB19, BD Biosciences), BV750 anti-human CD4 (Cat: 566355, Clone: SK3, BD Biosciences), BV570 anti-human CD8a (Cat: 301038, Clone: RPA-T8, BioLegend), Alexa Fluor 700 anti-human CD45RA (Cat: 304120, Clone: HI100, BioLegend), BV480 anti-human Tcrγδ (Cat: 566076, Clone: B1, BD Biosciences), BV605 anti-human CD27 (Cat: 356434, Clone: M-T271, BioLegend), PerCP-Cy5.5 anti-human CXCR3 (Cat: 353714, Clone: G025H7, BioLegend), PE-Cy7 anti-human CD197 (Cat: 557648, Clone: 3D12, BD Biosciences), BV785 anti-human CX3CR1 (Cat: 341628, Clone: 2A9-1, BioLegend), BV711 anti-human CXCR5 (Cat: 740737, Clone: RF8B2, BD Biosciences); intracellular markers included V 450 anti-human Granzyme B (Cat: 561151, Clone: GB11, BD Biosciences), PE anti-human FGFBP2 (Cat: 346603, Clone: TDA3, BioLegend).

### 2.5 Detection of myositis-specific autoantibodies

Eight myositis-specific autoantibodies (MSAs) were detected in patient serum using a transfected cell-based indirect immunofluorescence assay (cell-based assay, CBA). These antibodies included anti-MDA5, anti-Jo-1, anti-PL-7, anti-PL-12, anti-NXP2, anti-SAE1, anti-TIF1γ, and anti-Mi-2. The assay was performed using a commercial myositis eight-antibody detection kit (Guangzhou ImmunoArt Co., Guangzhou, China) according to the manufacturer’s instructions.

Briefly, mammalian cells expressing the corresponding target antigens and green fluorescent protein (GFP) as an internal control were fixed in 96-well plates. Each well was incubated with 80 μL of diluent containing 10% goat serum and 4 μL of patient serum at 37°C for 30 min. After washing, 80 μL of the fluorophore-conjugated secondary antibody diluted at 1:500 was added to each well. The plates were incubated at 37°C for 30 min in the dark. After additional washing steps, fluorescence signals were evaluated using an inverted fluorescence microscope. GFP-positive transfected cells were first identified using the green fluorescence channel. Specific fluorescence signals were then evaluated using the red fluorescence channel. A sample was considered positive for the corresponding antibody when the transfected cells showed clear red fluorescence, whereas non-transfected cells showed no detectable fluorescence or substantially weaker fluorescence. For patients with repeated blood sampling, autoantibody results were analyzed at the individual-patient level. Each patient was counted only once, using the earliest available stored serum sample.

### 2.6 Multiplex immunohistochemistry

Skin tissues were collected from Dermatomyositis patients at the Jiangnan University Medical Center. Representative lesion specimens were excised and immediately fixed. Immunofluorescence was performed on 3-μm serial sections of paraffin-embedded tissue following deparaffinization, antigen retrieval, and blocking of nonspecific interactions. These specimens were incubated with the primary antibodies: anti-CD4 (catalog ab183685, Abcam), anti-GZMB (catalog 13588-1-AP, PTG), CD31 (catalog ab182981, Abcam), cleaved caspase-3 (catalog AF7022, AFFinity) and HLA-DR (catalog 17221-1-AP, PTG). Secondary antibodies were used according to the species origin of the associated primary antibody. The samples were mounted with Antifade mounting Medium.

### 2.7 Microscopy and quantitative image analysis

For quantitative evaluation, the whole tissue section was scanned and converted into grayscale digital images across four fluorescence channels, including Cy3, Aqua, SPgreen, and SPorange, together with DAPI. Phenotypically defined cells were identified and enumerated using ImageJ (version 1.53k). Threshold values were established according to positive control samples. During tissue staining, DAPI served as a nuclear counterstain to identify nucleated cells, while specific markers were applied to characterize each cell population: endothelial cells (CD31^+^) and CD4^+^ cytotoxic T lymphocytes (CD4^+^, GZMB^+^).

### 2.8 Statistical analysis

Data are presented as mean ± standard deviation (SD). For comparisons between two groups, unpaired data were analyzed using the two-tailed Mann–Whitney U test or unpaired t test, and paired data were analyzed using the Wilcoxon matched-pairs signed-rank test or paired t test, as appropriate. When more than two groups were compared, the Kruskal–Wallis test followed by Dunn’s post hoc multiple comparison test or one-way ANOVA was applied. Baseline balance was assessed for sex, age, treatment status, and complications. Continuous variables were compared according to data distribution and the number of groups, while categorical variables were compared using the chi-square test or Fisher’s exact test. Associations between variables were evaluated using Pearson’s or Spearman’s correlation coefficients, as appropriate. Multivariable linear regression analyses were performed to assess the association of GZMB^+^CD4^+^ CTLs with MYOACT or CDASI-A scores after adjustment for sex, age, treatment status, and complications. All statistical analyses were conducted using GraphPad Prism version 10.1.2. A two-sided *P* value less than 0.05 was defined as indicating statistical significance.

## 3 Results

### 3.1 GZMB^+^CD4^+^ CTLs were expanded in the peripheral blood of patients with DM and ASS

**Figure 1:**
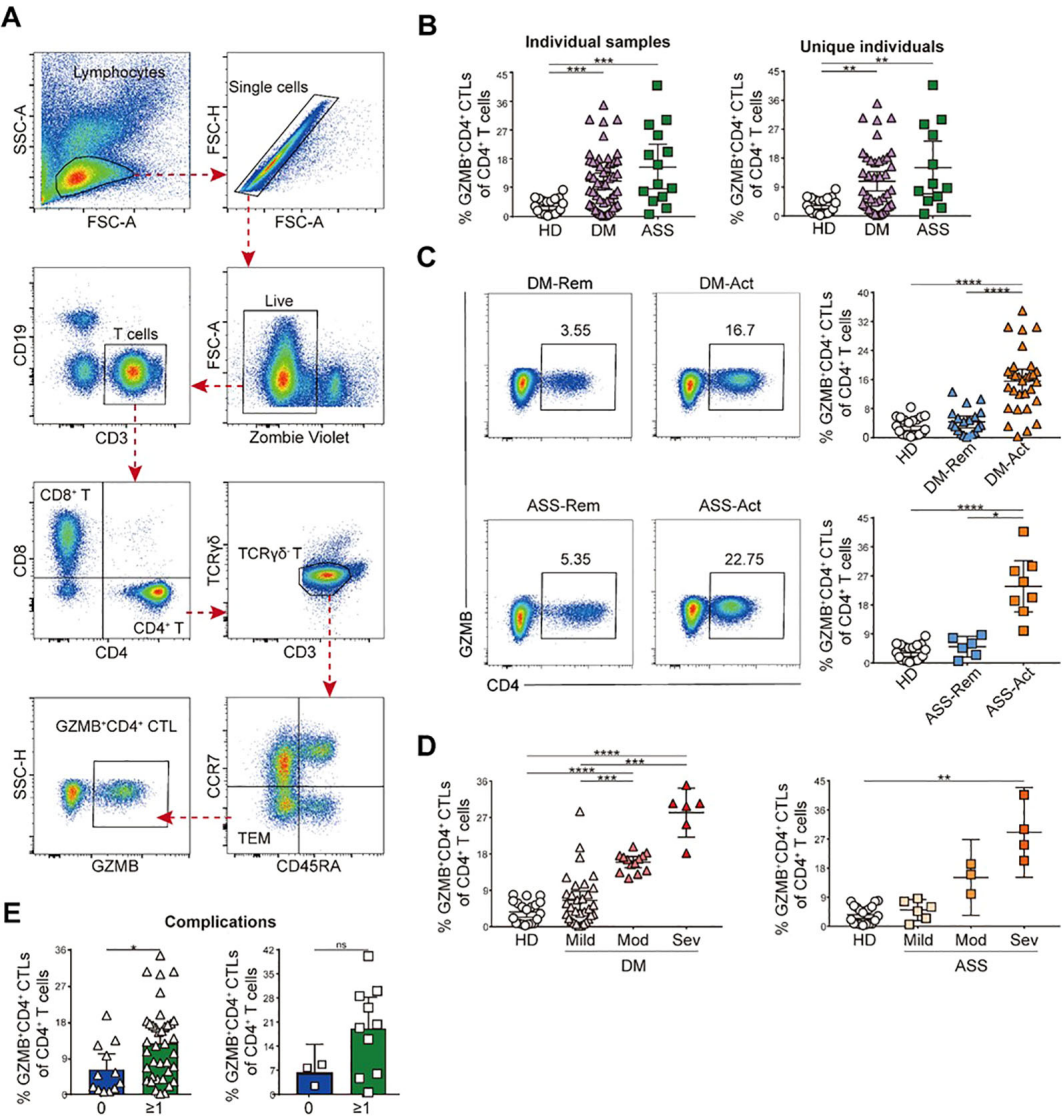
High proportions of GZMB^+^CD4^+^ CTLs are associated with patients in DM and ASS. **(A)** Gating strategy to identify GZMB^+^CD4^+^ CTLs by flow cytometry. **(B)** Left: proportion of GZMB^+^CD4^+^ CTLs among CD4^+^ T cells. Data from all the samples are shown. Right: proportion of GZMB^+^CD4^+^ CTLs among CD4^+^ T cells. When data were collected before and after treatment, only data from the samples before treatment are included here. **(C)** Left: representative flow cytometric plots showing the frequency of GZMB^+^CD4^+^ CTLs among CD4^+^ T cells from the indicated groups. Right: scatter plots showing the accumulated data for the frequency of GZMB^+^CD4^+^ CTLs from the indicated groups. **(D)** Scatter plots showing proportions of GZMB^+^CD4^+^ CTLs among CD4^+^ T cells in HD and in patients with DM and ASS classified as Mild, Moderate, and Severe. **(E)** Frequency of GZMB^+^CD4^+^ CTLs in DM (left) and ASS (right) patients without complications and with complications. *P < 0.05; **P < 0.01; ***P < 0.001; ****P < 0.0001; ns, not significant.

To examine the potential role of GZMB^+^CD4^+^ CTLs in the pathogenesis of DM and ASS, we first analyzed the levels of GZMB^+^CD4^+^ CTLs in peripheral blood samples from patients with DM and ASS by flow cytometry. Peripheral blood from HD and individuals with DM or ASS revealed that the effector memory T cells (dual loss of CCR7 and CD45RA) contained a population of GZMB^+^CD4^+^ CTLs. The flow cytometric gating strategy is shown in **Figure 1A**. Quantitative analysis revealed a significantly higher proportion of GZMB^+^CD4^+^ CTLs in both DM and ASS patients compared to HD (**Figure 1B**), whereas the frequency of conventional CD8^+^ CTLs remained comparable among these three groups (**Supplementary Figure 1**). As this study included data from 10 patients sampled before and after treatment, to exclude the potential influence of treatment interventions, we reassessed the dataset with post-treatment samples excluded (**Figure 1B**) The observed increase in GZMB^+^CD4^+^ CTLs remained extant after this adjustment. Next, we detected the distribution of GZMB^+^CD4^+^ CTLs in the HD group, the remission phase DM/ASS patient group (DM/ASS-Rem), and the active phase DM/ASS patient group (DM/ASS-Act) (**Figure 1C**). Of note, both DM-Act group and ASS-Act group patients exhibited significantly increased frequencies of GZMB^+^CD4^+^ CTLs compared with HD and patients in remission period.

Skin involvement is common in both DM and ASS. Therefore, we stratified the patients into mild, moderate (Mod), and severe (Sev) categories based on cutaneous disease activity (28). We then analyzed the proportion of GZMB^+^CD4^+^ CTLs in each group. Our analysis revealed a significant increase in the proportion of GZMB^+^CD4^+^ CTLs in the peripheral blood of DM and ASS patients correlating with the activity of skin lesions (**Figure 1D**). Subsequently, we investigated the correlation between GZMB^+^CD4^+^ CTLs and the presence of complications in DM and ASS patients. The analysis indicated that DM patients with one or more complications exhibited a significantly higher proportion of GZMB^+^CD4^+^ CTLs compared to those without complications. In contrast, no such difference was detected in ASS patients, potentially due to the limited sample size (**Figure 1E**).

In conclusion, our data demonstrate a significant expansion of the GZMB^+^CD4^+^ CTLs in the peripheral blood of DM and ASS patients. Moreover, we provide evidence that the variation in the proportion of these cells is associated with disease overall activity, skin activity, and complications, indicating that this T cell subset may play an important role in the pathological progression of the disease.

### 3.2 Phenotypic profiling of GZMB^+^CD4^+^ CTLs reveals tissue-homing capacity and enhanced cytotoxic potential in DM and ASS patients

**Figure 2:**
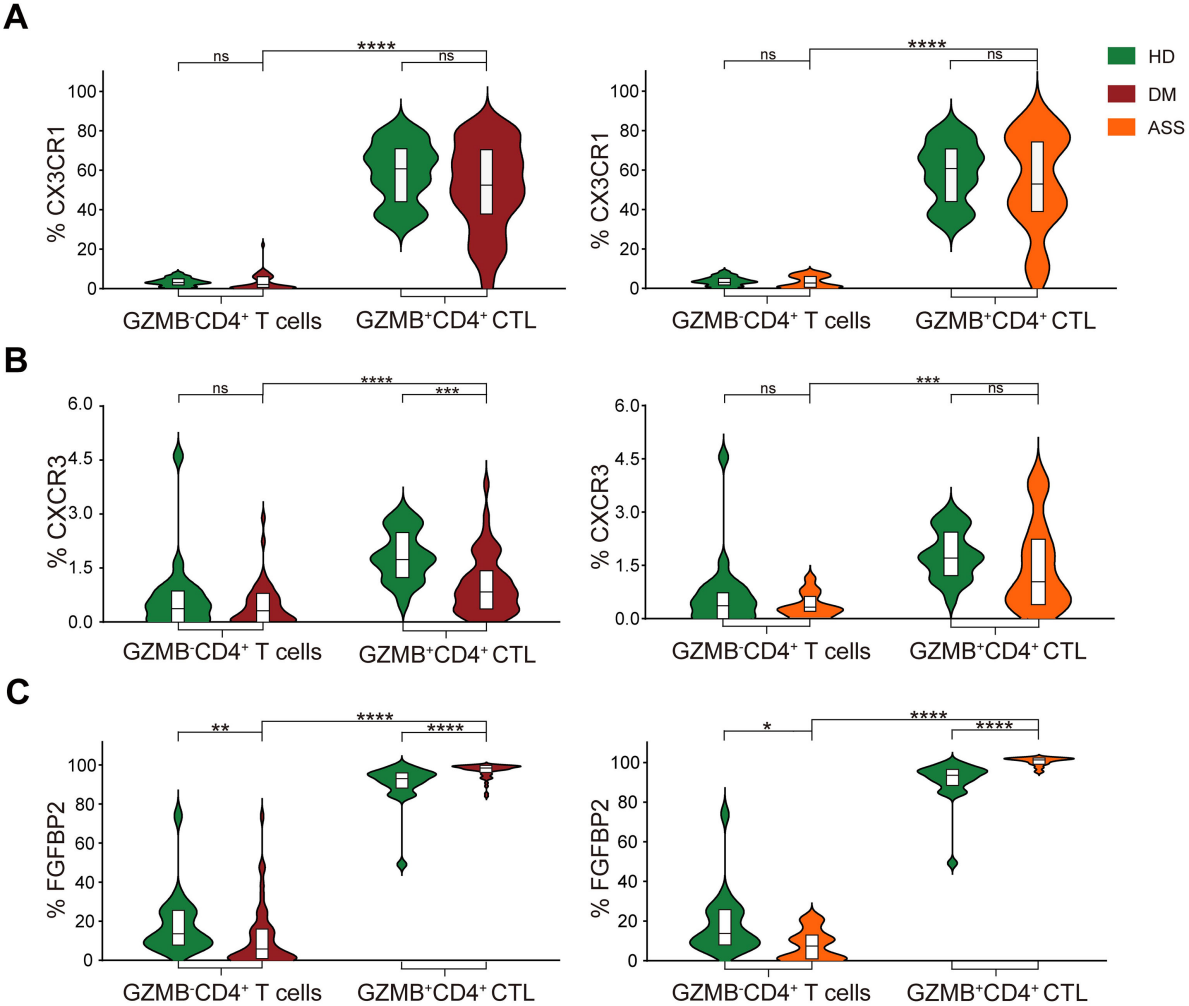
Frequency of markers associated with GZMB^+^CD4^+^ CTLs among CD4^+^ T cells. **(A)** Flow cytometry analysis of CX3CR1 expression in GZMB^-^CD4^+^ T cells compared with GZMB^+^CD4^+^ CTLs from the indicated groups. **(B)** Flow cytometry analysis of CXCR3 expression in GZMB^-^CD4^+^ T cells compared with GZMB^+^CD4^+^ CTLs from the indicated groups. **(C)** Intracellular flow cytometry analysis of FGFBP2 expression, showing higher frequency of this cytotoxic protein in GZMB^+^CD4^+^ T cells than in GZMB^-^CD4^+^ CTLs in DM and ASS patients. *P < 0.05; **P < 0.01; ***P < 0.001; ****P < 0.0001; ns, not significant.

The chemokine network is integral to the regulation of T cell infiltration into inflammatory sites. Previous research has indicated that C-X3-C motif chemokine ligand 1 (CX3CL1), the specific ligand for C-X3-C motif chemokine receptor 1 (CX3CR1), is markedly expressed in the affected muscle tissues from patients with polymyositis and DM (29), with elevated CX3CL1 levels potentially correlating with exacerbated muscle weakness in these patients. Furthermore, immunofluorescence staining of dermal tissues has demonstrated a substantial presence of CXCR3-positive cells within lymphocyte-infiltrated regions of the skin in dermatomyositis patients (29–31). Consequently, we assessed the chemotactic migration capacity of GZMB^+^CD4^+^ CTLs by analyzing the expression levels of CX3CR1 and CXCR3. A comparative analysis of CX3CR1 and CXCR3 expression between GZMB^+^CD4^+^ T cells and GZMB^-^CD4^+^ T cells revealed a significant enrichment of both CX3CR1 and CXCR3 in GZMB^+^CD4^+^ CTLs relative to their GZMB^-^CD4^+^ counterparts (**Figures 2A, B**). Notably, there was a trend of preferentially lower CX3CR1^+^ and CXCR3^+^ cells within the GZMB^+^CD4^+^ CTLs in DM patients compared with HD group, with similar trends observed in patients with ASS (**Figures 2A, B**). We hypothesize that the diminished proportions of CXCR3^+^GZMB^+^CD4^+^ CTLs and CX3CR1^+^GZMB^+^CD4^+^ CTLs in the peripheral blood of DM and ASS patients may result from their enhanced chemotactic activity, which facilitates the infiltration of these cell subsets into local lesion tissues.

To further elucidate the cytotoxic role of GZMB^+^CD4^+^ CTLs in individuals with DM and ASS, we assessed the expression levels of fibroblast growth factor binding protein 2 (FGFBP2), a critical effector molecule associated with cytotoxicity, utilizing flow cytometry. The findings revealed a significant upregulation of FGFBP2 expression in GZMB^+^CD4^+^ CTLs from both DM and ASS patients in comparison to HD (**Figure 2C**). Moreover, previous research has documented a downregulation of the co-stimulatory receptor CD27 in cytotoxic cells within the context of IgG4-related disease (32). In alignment with these observations, our study found that the majority of GZMB^+^CD4^+^ CTLs exhibited a loss of CD27 expression (**Supplementary Figure 2**). Collectively, these results suggest that GZMB^+^CD4^+^ CTLs possess enhanced tissue-homing capabilities and cytotoxic potential in patients with DM and ASS.

### 3.3 GZMB^+^CD4^+^ CTLs were highly correlated with the scores of global disease activity and skin disease activity in DM and ASS

**Figure 3:**
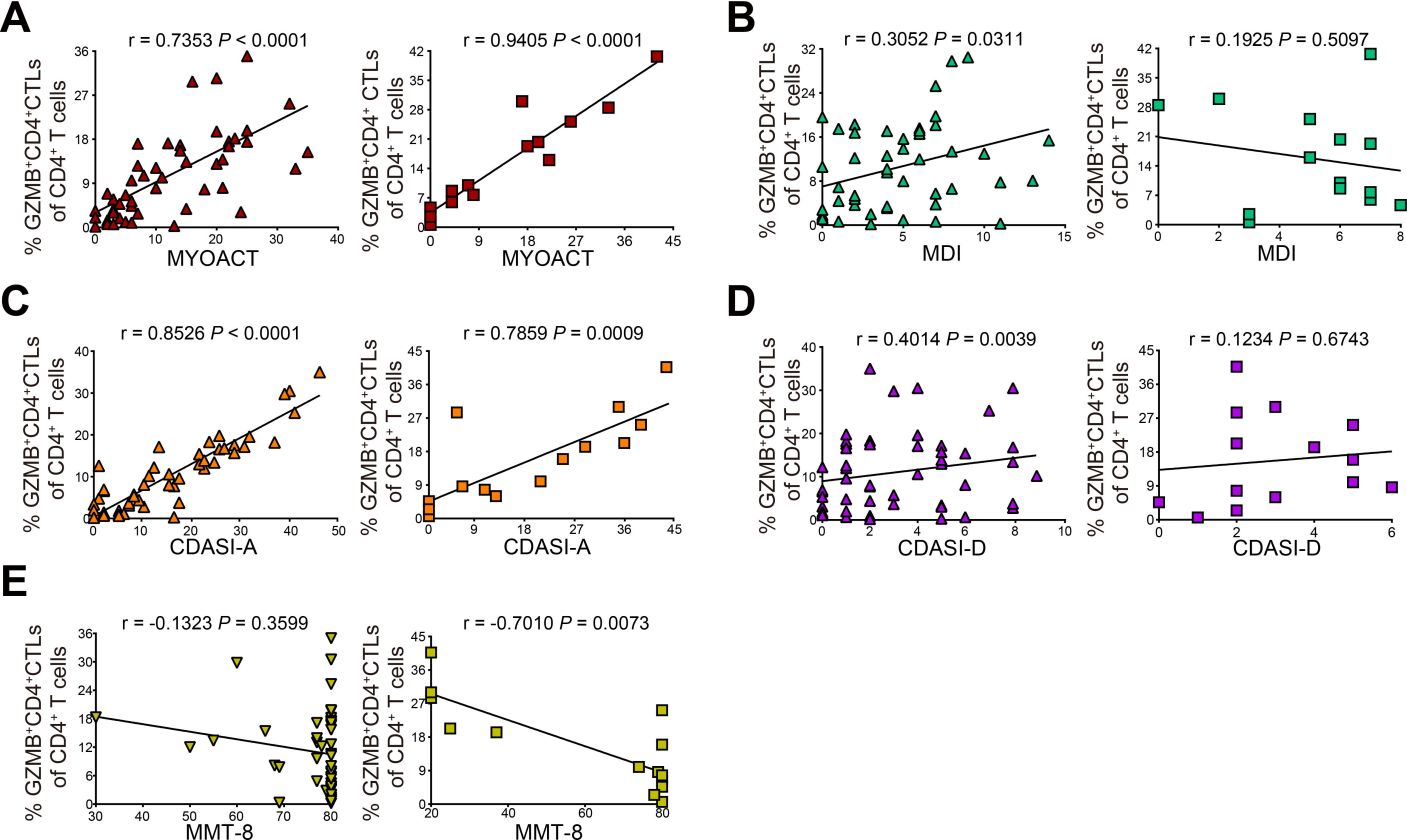
Peripheral proportion of GZMB^+^CD4^+^ CTLs is highly correlated with global disease activity and skin disease activity in DM and ASS. **(A–F)** Correlation between GZMB^+^CD4^+^ CTLs and measures of disease activity and severity, including the Myositis Disease Activity Assessment Tool (MYOACT) score **(B)**, Myositis Damage Index (MDI) score **(C)**, Cutaneous Dermatomyositis Disease Area and Severity Index activity (CDASI-A) score **(D)**, Cutaneous Dermatomyositis Disease Area and Severity Index damage (CDASI-D) score **(E)**, Manual Muscle Testing (MMT-8) score **(F)** in DM (left) and ASS (right) patients.

We further analyzed the relationships between the frequency of circulating GZMB^+^CD4^+^ CTLs and standardized clinical assessment scores of MYOACT, MDI, CDASI, and MMT-8. We found that the percentage of GZMB**^+^**CD4**^+^** CTLs was positively correlated with MYOACT score which evaluated global disease activity, in both DM (Spearman’s r = 0.7353, *P* < 0.0001) and ASS (Spearman’s r = 0.9405, *P* < 0.0001) patients (**Figure 3A**). In Sjogren’s syndrome, CD4^+^ CTLs correlated positively with disease activity, which is similar to our study (33). The proportion of GZMB^+^CD4^+^ CTLs displayed a weak positive association with MDI score, a measure reflecting global disease damage, in DM patients, but no significant relationship was identified with MDI score in ASS patients (**Figure 3B**). Similarly, the frequency of GZMB^+^CD4^+^ CTLs correlated strongly with skin disease activity, as assessed by the CDASI-A score, in both DM (Spearman’s r = 0.8526, *P* < 0.0001) and ASS (Pearson’s r = 0.7859, *P* = 0.0009; **Figure 3C**). The percentage of GZMB^+^CD4^+^ CTLs displayed a weak positive association with CDASI-D score, a measure reflecting skin damage, in DM patients, but no significant relationship was identified with CDASI-D score in ASS patients (**Figure 3D**). We also evaluated relationship between GZMB^+^CD4^+^ CTLs with muscle strength. Of note, we observed a significant negative correlation between the frequency GZMB^+^CD4^+^ CTLs and MMT-8 score in ASS patients (Spearman’s r = -0.7010, *P* = 0.0073; **Figure 3E**). In contrast, DM patients exhibited no such correlation (Spearman’s r = -0.1323, *P* = 0.3599), which may be partly explained by the narrow distribution of MMT-8 scores in this subgroup, as most DM patients had preserved or only slightly reduced muscle strength.

To account for potential clinical confounding, we assessed the baseline balance of sex, age, treatment status, and complications across active/remission groups and CDASI-A activity subgroups in patients with DM and ASS. No significant imbalance was observed between active and remission groups in either disease group (**Supplementary Table 3**). Across CDASI-A activity subgroups, age and treatment status differed among DM subgroups, whereas no significant differences were observed among ASS subgroups (**Supplementary Table 4**). Accordingly, sex, age, treatment status, and complications were included as covariates in multivariable linear regression models using MYOACT or CDASI-A scores as dependent variables. After adjustment, GZMB^+^CD4^+^ CTLs remained significantly associated with both scores in patients with DM and ASS (**Supplementary Table 5**).

In summary, we observed that the proportion of GZMB^+^CD4^+^ CTLs is highly correlated with global disease activity and skin disease activity in patients with DM and ASS.

### 3.4 GZMB^+^CD4^+^ CTLs correlate with treatment response independently of specific drug regimens

**Figure 4:**
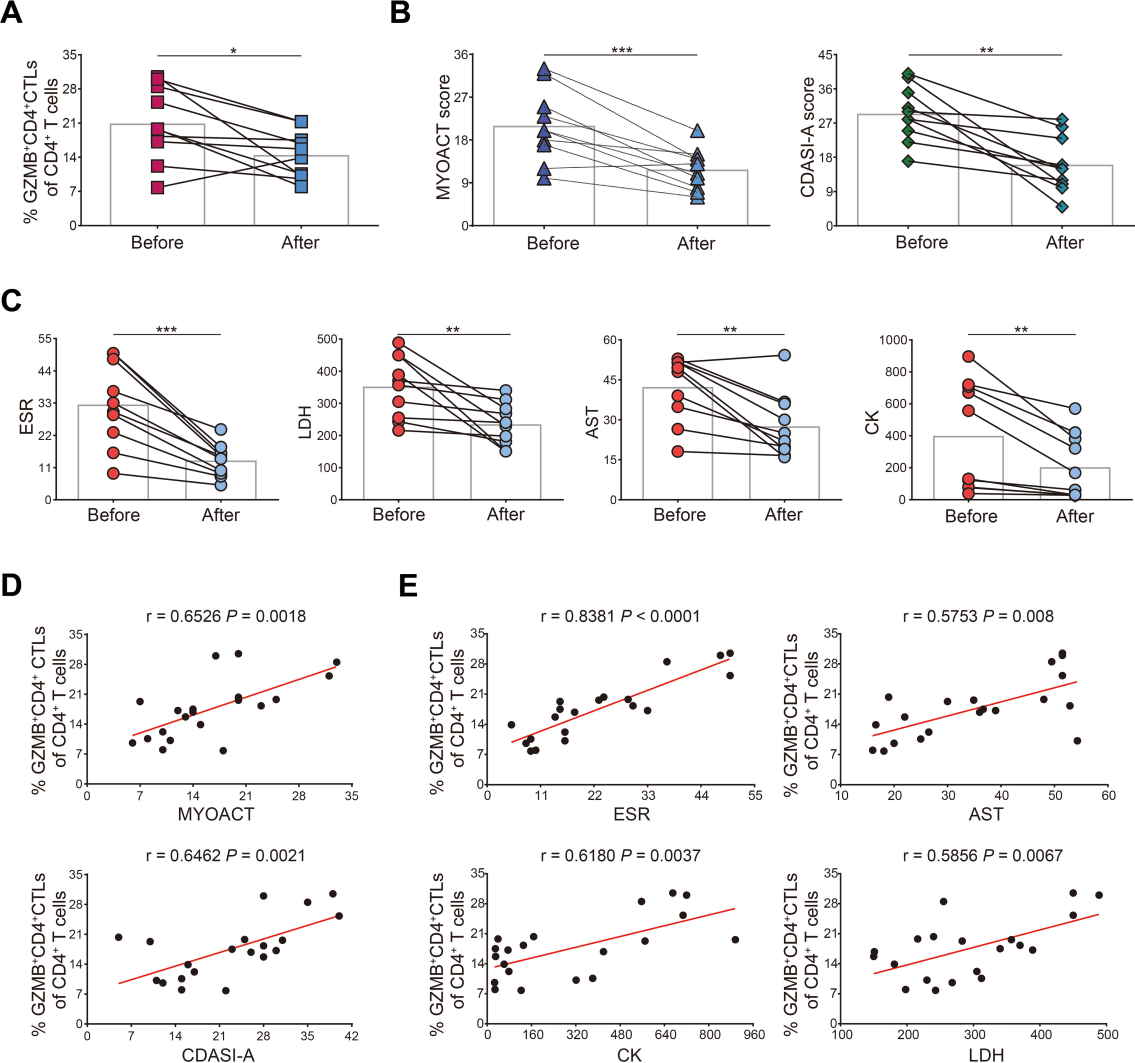
GZMB^+^CD4^+^ CTLs decline after treatment and are associated with disease activity in IIM patients. **(A)** Frequency of GZMB^+^CD4^+^ CTLs among CD4^+^ T cells in paired peripheral blood samples from IIM patients (n=10) before and after treatment. **(B)** MYOACT and CDASI-A scores in the same patients before and after treatment. **(C)** Laboratory observations of systemic inflammation and muscle damage (ESR, LDH, AST, CK) before and after treatment. **(D)** Correlations between GZMB^+^CD4^+^ CTLs and MYOACT or CDASI-A scores (Including samples before and after treatment). **(E)** Correlation between GZMB^+^CD4^+^ CTLs and laboratory parameters (ESR, LDH, AST, CK), using paired pre- and post-treatment samples. *P < 0.05; **P < 0.01; ***P < 0.001; ****P < 0.0001; ns, not significant.

To evaluate the potential of peripheral GZMB^+^CD4^+^ CTLs in monitoring the clinical therapeutic response, paired blood samples were collected from ten IIM patients before and after treatment. The specific therapeutic regimens employed are detailed in **Supplementary Table 6**. Following treatment, the proportion of GZMB^+^CD4^+^ CTLs was significantly reduced (**Figure 4A**). Concurrently, disease activity-related clinical scores and laboratory markers showed significant improvement after treatment. These included the MYOACT score, CDASI-A score, erythrocyte sedimentation rate (ESR), lactate dehydrogenase (LDH), aspartate aminotransferase (AST), and creatine kinase (CK) (**Figures 4B, C**). These changes indicated effective alleviation of clinical symptoms. To further explore the utility of GZMB^+^CD4^+^ CTLs in monitoring therapeutic efficacy, we analyzed the correlation between changes in these cells and the aforementioned clinical indicators. The results revealed a significant positive correlation between the proportion of GZMB^+^CD4^+^ CTLs and the MYOACT score, CDASI-A score, ESR, LDH, AST, and CK (**Figures 4D, E**).

To determine whether the observed decrease in GZMB^+^CD4^+^ CTLs was a direct consequence of specific medications, we analyzed their levels in the context of different therapeutic regimens. The majority of patients included in the study were administered glucocorticoid (GC) therapy (**Supplementary Table 7**). Our findings indicated that there were no significant differences in the proportions of GZMB^+^CD4^+^ CTLs among patients receiving treatment with GC, hydroxychloroquine (HCQ), mycophenolate mofetil (MMF) and tripterygium glycosides (TG; **Supplementary Figure 3**). Overall, despite the variability in therapeutic regimens, no significant alterations in the proportion of GZMB^+^CD4^+^ CTLs were attributed to specific drugs. This suggests that the observed reduction in these cells following drug treatment likely reflects effective disease amelioration rather than a direct pharmacological effect.

### 3.5 GZMB^+^CD4^+^ CTLs infiltrated in DM skin lesions

**Figure 5:**
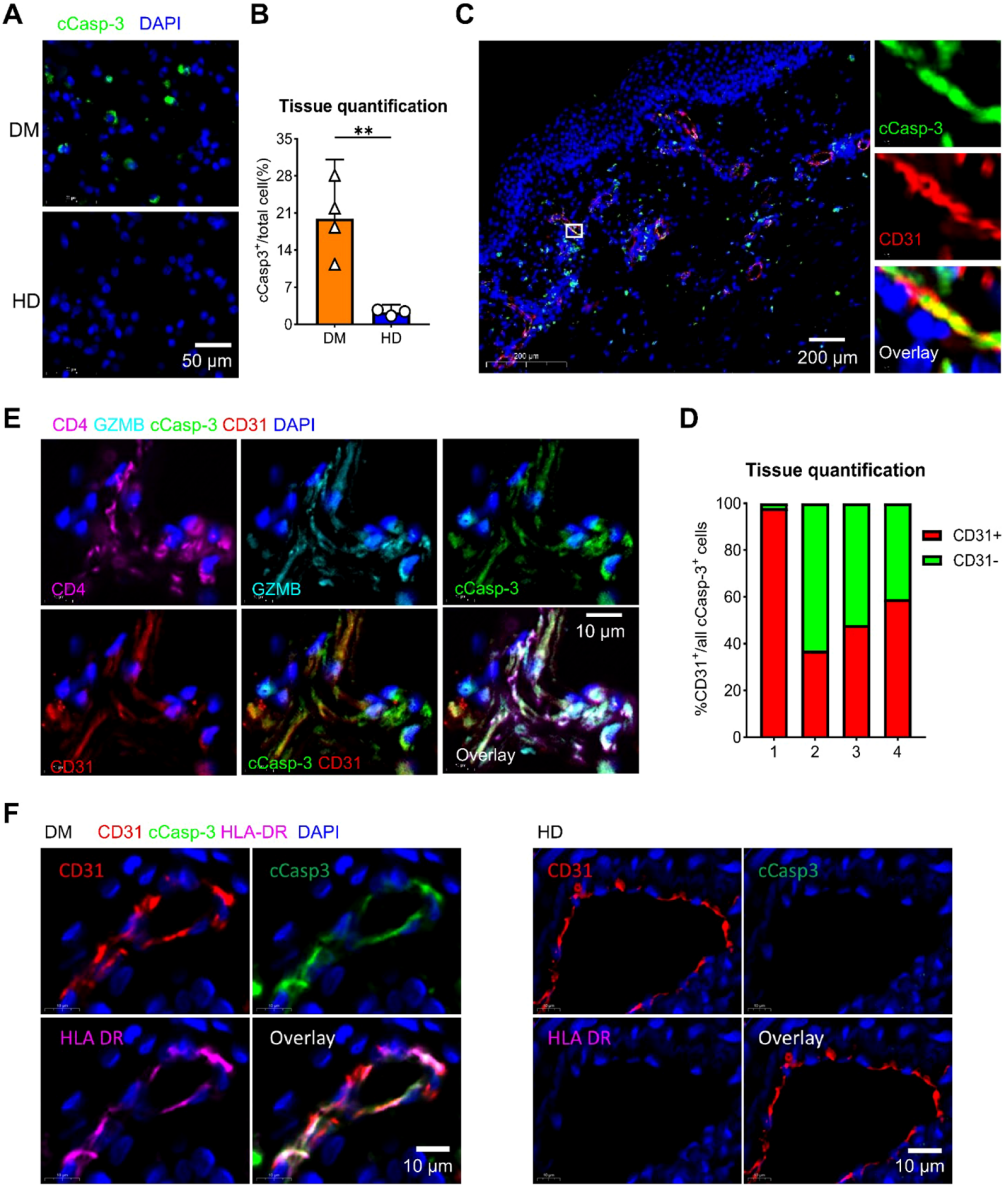
Endothelial cells expressing HLA class II are frequent targets of apoptosis in DM tissues. **(A)** Representative multiplex immunohistochemistry images showing cleaved caspase-3 (cCasp-3) staining (green) in DM (n=4) and control skin (n=3) samples. **(B)** Proportions of cCasp-3^+^ apoptotic cells in DM patients and control skin. **(C)** Representative multiplex immunohistochemistry images showing CD31-expressing endothelial cells (red) with cCasp-3 staining (green) in DM skin tissue. **(D)** Relative proportions of apoptotic cCasp-3^+^ endothelial cells (red) and nonendothelial apoptotic cells (green) in DM tissues. **(E)** Representative multiplex immunohistochemistry image showing CD4^+^ (pink) GZMB^+^ (aqua) and cCasp-3^+^ (green) endothelial cells (red) in DM tissue. **(F)** Multiplex immunohistochemistry images of cCasp-3^+^ (green) and HLA-DR^+^ (pink) endothelial cells (red) in DM, but not in control skin. **P < 0.01.

Given that microvascular injury, endothelial activation, and impaired endothelial regeneration are recognized features of DM lesions, we focused on CD31^+^ endothelial cells to assess their potential association with tissue-infiltrating GZMB^+^CD4^+^ CTLs (34–38). To explore the potential for host cell targeting by tissue-infiltrating GZMB^+^CD4^+^ CTLs, apoptotic cells were quantified using an anti-cleaved caspase-3 antibody (cCasp-3) (**Figure 5A**). Consistent with observations in systemic sclerosis (39), DM lesional tissues exhibited a significantly higher number of apoptotic cells relative to HD controls (**Figure 5B**). This was accompanied by a marked increase in the proportion of apoptotic endothelial cells (cCasp3-3^+^CD31^+^), which constituted more than 50% of apoptotic cells in certain lesions (**Figures 5C, D**). Notably, GZMB^+^CD4^+^ CTLs abundantly infiltrated in DM skin lesions (**Figure 5E**).

Given that CD4^+^ CTLs typically mediate cytotoxicity through an HLA class II-restricted recognition, we hypothesized that endothelial cells in DM patients might upregulate HLA class II molecules, rendering them susceptible to recognition by HLA class II-restricted TCRs on CD4^+^ CTLs. Indeed, HLA-DR expression was significantly upregulated on endothelial cells in DM patients compared to HD, and some HLA-DR^+^ endothelial cells were also positive for cCasp-3. These findings suggest that HLA class II-mediated antigen presentation, in conjunction with the accumulated GZMB^+^CD4^+^ CTLs, contributes to endothelial cell apoptosis in DM (**Figure 5F**).

### 3.6 GZMB^+^CD4^+^ CTLs may serve as a potential biomarker for assessing disease activity

**Figure 6:**
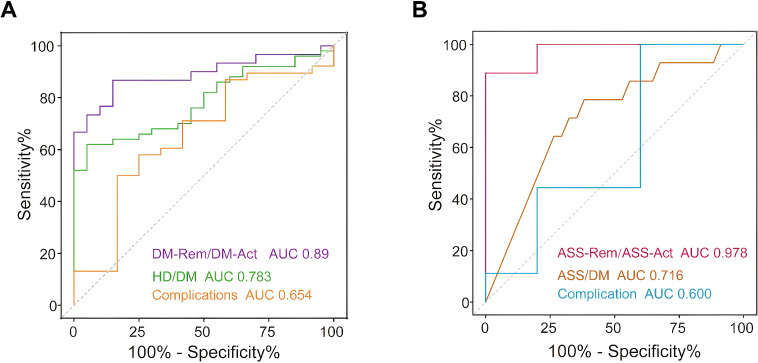
Receiver operating characteristic (ROC) analysis of the indicated parameters to diagnose, identify active patients and predict complications in DM **(A)** and ASS **(B)** patients.

To evaluate the clinical utility of GZMB^+^CD4^+^ CTLs in DM and ASS, we performed a receiver operating characteristic (ROC) curve analysis. The results showed that GZMB^+^CD4^+^ CTLs could effectively distinguish patients with DM or ASS from healthy individuals. The optimal diagnostic cutoff value was determined to be 6.3% for DM (area under the curve [AUC] = 0.783; 95% confidence interval [CI]: 0.678–0.888; **Figure 6A**) and 5.9% for ASS (AUC = 0.716; 95% CI: 0.557–0.876; see **Figure 6B**). At these thresholds, the proportion of GZMB^+^CD4^+^ CTLs effectively distinguished patients from HD, yielding a sensitivity of 83.3% and specificity of 50% for DM (**Figure 6A**), and a sensitivity of 100% and specificity of 40% for ASS (**Figure 6B**).

We next assessed the capacity of GZMB^+^CD4^+^ CTLs to stratify disease activity. Using a cutoff of 7.9%, these cells effectively differentiated between active and remitting DM (AUC = 0.890; 95% CI: 0.798–0.982). In ASS, GZMB^+^CD4^+^ CTLs exhibited even stronger discriminatory performance (AUC = 0.978; 95% CI: 0.910–1.000). Collectively, these findings suggest that GZMB^+^CD4^+^ CTLs may serve as a reliable biomarker for identifying active disease and assessing the risk of clinical progression.

## 4 Discussion

Traditionally, CD4^+^ T cells were classified as helper T cells with immunomodulatory functions, while CD8^+^ T cells were considered the primary cytotoxic effectors. This classical view, however, has been refined by recent discoveries. It is now clear that certain CD4^+^ T cell subsets acquire cytotoxic potential, expressing granzymes and perforin to kill target cells in an antigen-specific, MHC class II-restricted manner (40–42). These cells, designated CD4^+^ CTLs, play protective roles in viral and tumor clearance but are associated with pathogenic effects in autoimmune diseases. In active IgG4-related disease, clonally expanded GZMA^+^PRF1^+^CD4^+^ CTLs accumulated in peripheral blood and affected tissues. And glucocorticoid-induced remission was associated with a decrease of these cells (43). In systemic sclerosis, GZMA^+^CD4^+^ T cells infiltrate skin lesions and colocalize with apoptotic HLA-DR^+^ endothelial cells, implicating them in direct cytotoxic tissue damage (39). Moreover, an expanded population of GZMB^+^Eomes^+^ CD4^+^ CTLs in multiple sclerosis has been linked to disease progression, with Eomes expression serving as a potential biomarker (44).

In this study, we identify a previously uncharacterized subset of GZMB^+^CD4^+^ cytotoxic T lymphocytes (CTLs) that is expanded in DM and ASS. Unlike GZMB^+^CD8^+^ CTLs, this GZMB^+^CD4^+^ CTL subset was significantly increased in the peripheral blood of both patient groups and was prominently enriched in DM skin lesions. Notably, the frequency of this subset was significantly elevated during active disease and declined following standard glucocorticoid or immunosuppressive therapy, suggesting a close association with disease activity. There was a significant positive correlation with clinical activity scores (MYOACT and CDASI-A), but not with cumulative damage indices (MDI and CDASI-D). These findings suggest that GZMB^+^CD4^+^ CTLs expand rapidly during disease exacerbation and may actively contribute to pathogenesis via cytotoxic mechanisms.

Given that the expression of the chemokine receptor CX3CR1 defines GZMB^+^CD4^+^ T cells in both dengue virus infection and colorectal cancer (45, 46), we sought to investigate this association in DM and ASS. We confirmed enrichment of CX3CR1 in GZMB^+^ versus GZMB^-^CD4^+^ T cells, although the frequency of CX3CR1^+^ cells within the GZMB^+^ subset trended lower compared to healthy group. Circulating GZMB^+^CD4^+^ CTLs from DM and ASS patients also showed reduced CXCR3 expression. Our finding is in line with previous study showing that elevated serum levels of CXCL10—a primary ligand for CXCR3—in DM patients, which inversely correlated with the frequency of circulating CXCR3^+^CD4^+^ T cells (9). These data suggest that the CXCL10-CXCR3 axis may be associated with the migration of CXCR3^+^CD4^+^ CTLs from the periphery to inflamed tissues. Indeed, we found abundant CD4^+^ CTLs in DM skin lesions, which were spatially associated with apoptotic endothelial cells. These endothelial cells also showed increased HLA-DR expression. Given that CD4^+^ CTLs exert their cytotoxic effects in an HLA class II-restricted manner, these findings suggest that endothelial cells may serve as potential targets of tissue-infiltrating GZMB^+^CD4^+^ CTLs in DM skin lesions. In addition, our flow cytometric analysis primarily assessed the proportion of GZMB^+^CD4^+^ CTLs among CD4^+^ T cells rather than their absolute cell counts. Therefore, the increased frequency of GZMB^+^CD4^+^ CTLs observed in active disease may reflect a relative enrichment of this subset, but may also be influenced by changes in other CD4^+^ T cell populations. Future studies incorporating absolute cell counts and broader profiling of CD4^+^ T cell subsets will be required to clarify this issue.

This study has several limitations. First, the relatively small sample size of the antisynthetase syndrome (ASS) patient cohort—due to constraints in patient recruitment—limited our ability to comprehensively evaluate the role of GZMB^+^CD4^+^ CTLs in this disease subtype. Therefore, findings related to ASS should be interpreted with caution. Second, although DM and ASS were classified according to clinical diagnosis and predominant clinical phenotype, potential overlap between IIM phenotypes could not be completely excluded. Third, the multiplex immunohistochemistry analysis was based on a limited number of skin biopsy samples, including four DM samples and three controls. These tissue-level findings should therefore be considered preliminary and require validation in larger cohorts. Fourth, the single-center design of this study may introduce selection bias and limit generalizability of our findings. In addition, this study lacked validation in an independent external cohort. Therefore, future multicenter studies with larger sample sizes and independent external validation cohorts are needed to further validate the role and clinical relevance of GZMB^+^CD4^+^ CTLs across diverse patient groups.

In conclusion, we report a significant expansion of GZMB^+^CD4^+^ T cells in the peripheral blood of patients with DM and ASS, accompanied by enhanced cytotoxic function. The frequency of this cell subset correlates closely with disease activity. Our mechanistic data suggest these cells are recruited to inflamed tissues, where they interact with HLA-DR^+^ endothelial cells and contribute to endothelial apoptosis, thereby driving tissue injury. More importantly, by uncovering this previously underappreciated cell subset, our work advances the mechanistic understanding of these myopathies and supports further exploration of GZMB^+^CD4^+^ CTLs as a potential therapeutic target.

## Data Availability

The raw data supporting the conclusions of this article will be made available by the authors, without undue reservation.

## Supplementary material

Supplementary Figure 1 Proportions of GZMB^+^CD8^+^ CTLs are associated with patients in DM and ASS. **(A)** Proportion of GZMB^+^CD8^+^ CTLs among CD8^+^ T cells. Data from all samples are shown. **(B)** Proportion of GZMB^+^CD8^+^ CTLs among CD8^+^ T cells. When data were obtained at multiple times, only data from the first sample are included here. **P* < 0.05; ***P* < 0.01; ****P* < 0.001; *****P* < 0.0001; ns, not significant.

Supplementary Figure 2 Expression of CD27 on GZMB^+^CD4^+^ CTL. **(A)** Surface CD27 expression on GZMB^+^CD4^+^ CTLs was assessed by t-SNE analysis and flow cytometry. **(B)** Pie chart showing the mean frequency of CD27 expression on GZMB^+^CD4^+^ CTLs, calculated from all HD and IIM patients.

Supplementary Figure 3 Relationship between treatment and the level of GZMB^+^CD4^+^ CTLs Scatter plots show the percentages of GZMB^+^CD4^+^ CTLs in DM (left) and ASS (right) patients treated with glucocorticoid (GC), hydroxychloroquine (HCQ), mycophenolate mofetil (MMF), and tripterygium glycosides (TG). No significant differences were observed among the groups.
