# Supplementary figures and images for: Dermatomyositis and antisynthetase syndrome disease activity is associated with the expansion of peripheral GZMB+CD4+ cytotoxic T cells
Source: Front Immunol. 2026 Jul 6;17:1863690. doi: 10.3389/fimmu.2026.1863690 (PMC13381829; doi:10.3389/fimmu.2026.1863690)

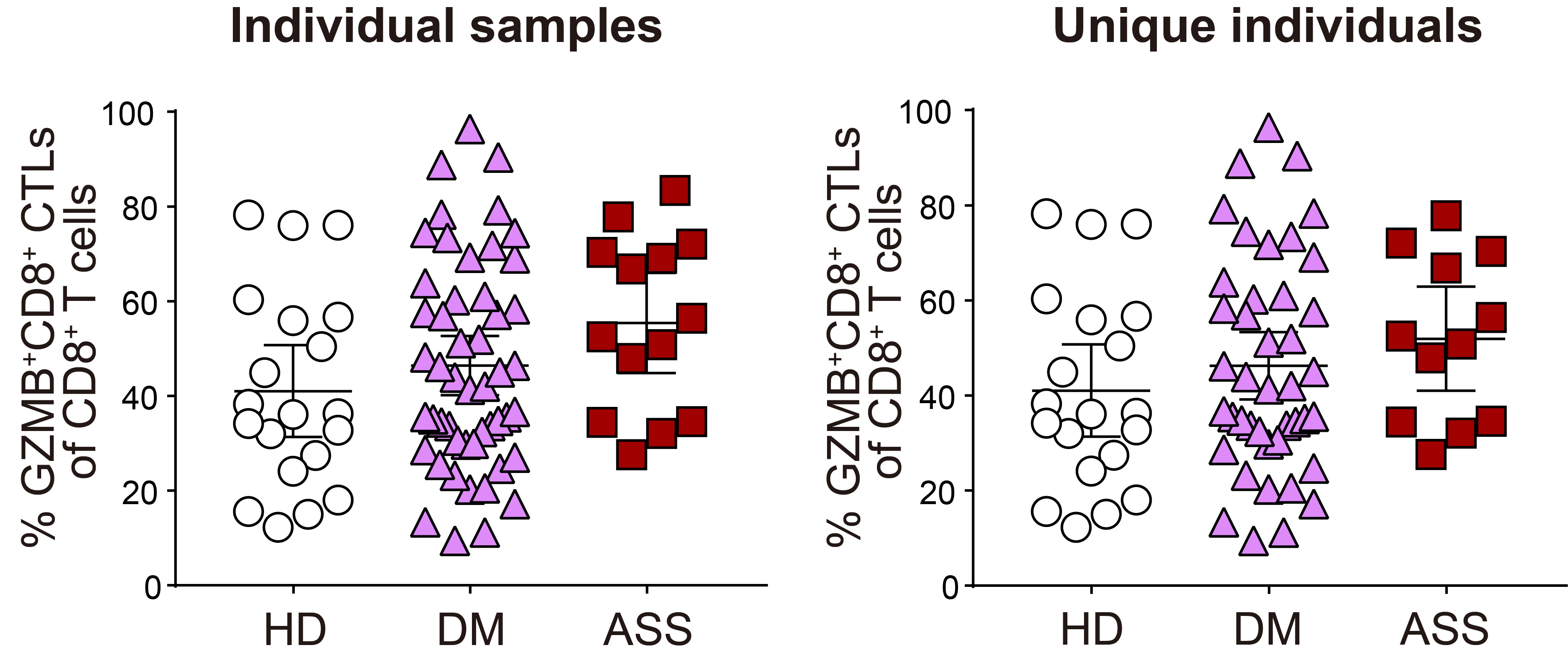

Supplement: Supplementary Figure 1 — Proportions of GZMB+CD8+ CTLs are associated with patients in DM and ASS. (A) Proportion of GZMB+CD8+ CTLs among CD8+ T cells. Data from all samples are shown. (B) Proportion of GZMB+CD8+ CTLs among CD8+ T cells. When data were obtained at multiple times, only data from the first sample are included here. *P < 0.05; **P < 0.01; ***P < 0.001; ****P < 0.0001; ns, not significant. [file Image1.jpeg]

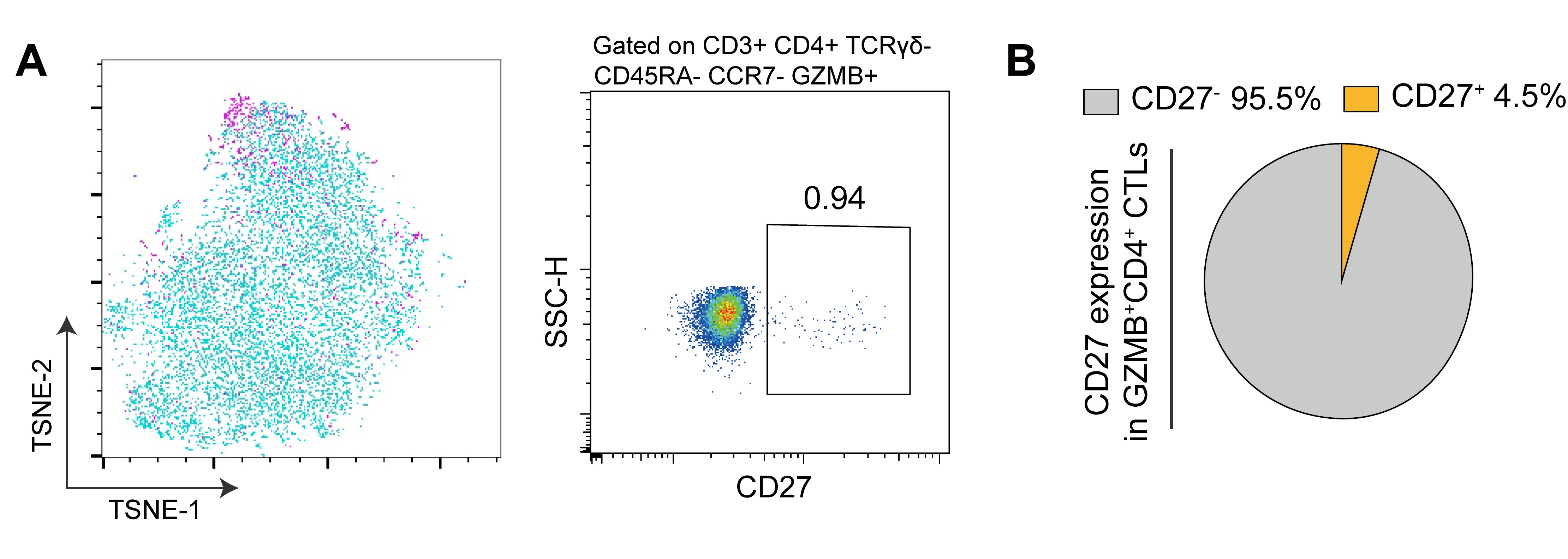

Supplement: Supplementary Figure 2 — Expression of CD27 on GZMB+CD4+ CTL. (A) Surface CD27 expression on GZMB+CD4+ CTLs was assessed by t-SNE analysis and flow cytometry. (B) Pie chart showing the mean frequency of CD27 expression on GZMB+CD4+ CTLs, calculated from all HD and IIM patients. [file Image2.jpeg]

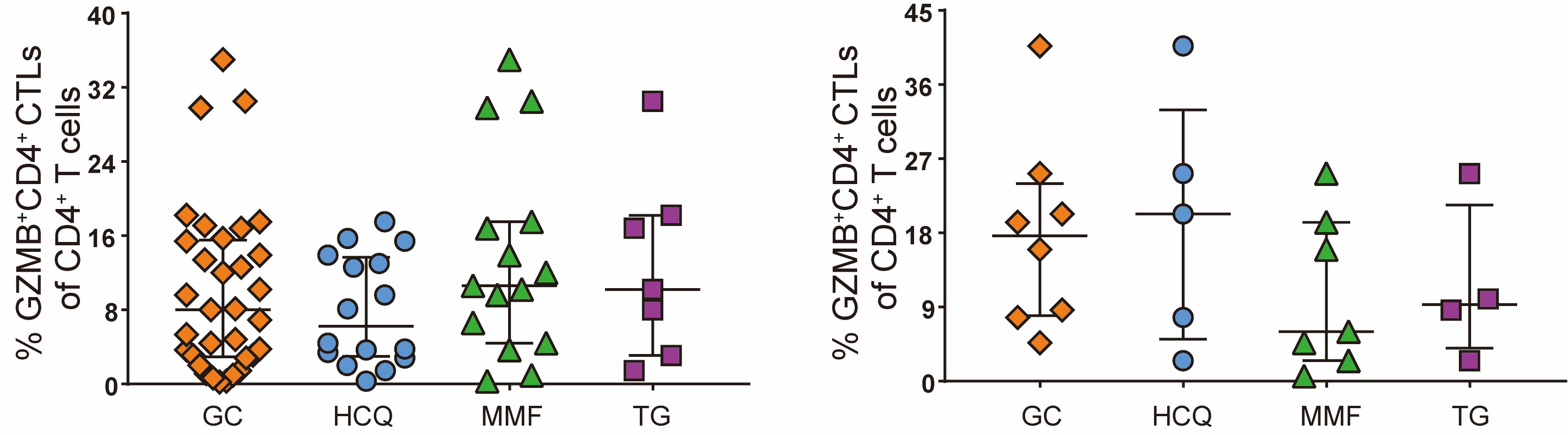

Supplement: Supplementary Figure 3 — Relationship between treatment and the level of GZMB+CD4+ CTLs Scatter plots show the percentages of GZMB+CD4+ CTLs in DM (left) and ASS (right) patients treated with glucocorticoid (GC), hydroxychloroquine (HCQ), mycophenolate mofetil (MMF), and tripterygium glycosides (TG). No significant differences were observed among the groups. [file Image3.jpeg]
